# Local singular characteristics on $\mathbb {R}^2$

**DOI:** 10.1007/s40574-021-00279-4

**Published:** 2021-02-22

**Authors:** Piermarco Cannarsa, Wei Cheng

**Affiliations:** 1 Dipartimento di Matematica, Università di Roma “Tor Vergata”, Via della Ricerca Scientifica 1, 00133 Rome, Italy; 2 Department of Mathematics, Nanjing University, Nanjing, 210093 China

**Keywords:** Hamilton–Jacobi equation, Viscosity solution, Singular characteristics, 35F21, 49L25, 37J50

## Abstract

The singular set of a viscosity solution to a Hamilton–Jacobi equation is known to propagate, from any noncritical singular point, along singular characteristics which are curves satisfying certain differential inclusions. In the literature, different notions of singular characteristics were introduced. However, a general uniqueness criterion for singular characteristics, not restricted to mechanical systems or problems in one space dimension, is missing at the moment. In this paper, we prove that, for a Tonelli Hamiltonian on $\mathbb {R}^2$, two different notions of singular characteristics coincide up to a bi-Lipschitz reparameterization. As a significant consequence, we obtain a uniqueness result for the class of singular characteristics that was introduced by Khanin and Sobolevski in the paper [On dynamics of Lagrangian trajectories for Hamilton-Jacobi equations. *Arch. Ration. Mech. Anal.*, 219(2):861–885, 2016].

## Introduction

This paper is devoted to study the local propagation of singularities for viscosity solutions of the Hamilton–Jacobi equations 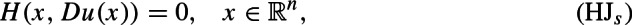

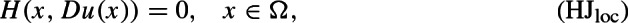


where *H* is a Tonelli Hamiltonian in ($\hbox {HJ}_s$) and *H* is of class $C^1$ and strictly convex in the *p*-variable in ($\hbox {HJ}_{\mathrm{loc}}$). In ($\hbox {HJ}_s$), we assume that 0 on the right-hand side is Mañé’s critical value. The existence of global weak KAM solutions of ($\hbox {HJ}_s$) was obtained in [12]. In ($\hbox {HJ}_{\mathrm{loc}}$), we suppose $\Omega \subset \mathbb {R}^n$ is a bounded domain.

Semiconcave functions are nonsmooth functions that play an important role in the study of ($\hbox {HJ}_s$) and ($\hbox {HJ}_{\mathrm{loc}}$). For semiconcave viscosity solutions of Hamilton–Jacobi equations, Albano and the first author proved in [1] that singular arcs can be selected as generalized characteristics. Recall that a Lipschitz arc $\mathbf {x}:[0,\tau ]\rightarrow \mathbb {R}^n$ is called a *generalized characteristic* starting from *x* for the pair (*H*, *u*) if it satisfies the following:

1.1 $$\begin{aligned} {\left\{ \begin{array}{ll} \dot{\mathbf {x}}(s)\in \mathrm {co}\,H_p\big (\mathbf {x}(s),D^+u(\mathbf {x}(s))\big )&{}\quad \text {a.e.}\;s\in [0,\tau ],\\ \dot{\mathbf {x}}(0)=x,&{} \end{array}\right. } \end{aligned}$$

where $\mathrm {co}$ stands for the convex hull. If $x\in \text{ Sing }\,(u)$—the singular set of *u*—then [1, Theorem 5] gives a sufficient condition for the existence of a generalized characteristic propagating the singularity of *u* locally.

The local structure of singular (generalized) characteristics was further investigated by the first author and Yu in [11], where *singular characteristics* were proved more regular near the starting point than the arcs constructed in [1]. Such additional properties will be crucial for the analysis we develop in this paper.

For any weak KAM solution *u* of ($\hbox {HJ}_s$), the class of *intrinsic singular* (generalized) *characteristics* was constructed in [4] by the authors of this paper, using the positive type Lax-Oleinik semi-group. Such a method allowed to construct global singular characteristics, which we now call *intrinsic*. Moreover, in [5, 6] the “intrisic approach” turned out to be useful for pointing out topological properties of the cut locus of *u*, including homotopy equivalence to the complement of the Aubry set (see also [7] for applications to Dirichlet boundary value problems).

In spite of its success in capturing singular dynamics, it could be argued that the relaxation procedure in the original definition of generalized characteristics—that is, the presence of the convex hull in (1.1)—might cause a loss of information coming from the Hamiltonian dynamics behind. On the other hand, such a relaxation is necessary to ensure convexity of admissible velocities for the differential inclusion in (1.1), since the map $x\rightrightarrows H_p(x,D^+u(x))$ fails to be convex-valued, in general.

The most important example where the above relaxation is unnecessary is probably given by mechanical Hamiltonians of the form $H(x,p)=\frac{1}{2}\langle A(x)p,p\rangle +V(x)$, where *A*(*x*) is a symmetric positive definite $n\times n$-matrix smoothly depending on *x* and *V*(*x*) is a smooth function on $\mathbb {R}^n$. In this case, (1.1) reduces to the *generalized gradient system*

1.2 $$\begin{aligned} {\left\{ \begin{array}{ll} \dot{\mathbf {x}}(t)\in A(\mathbf {x}(t))D^+u(\mathbf {x}(t))&{}\quad t>0\quad \text {a.e.}\\ \dot{\mathbf {x}}(0)=x,&{} \end{array}\right. } \end{aligned}$$

the solution of which, unique for any initial datum, forms a Lipschitz semi-flow (see, e.g., [1–3, 8, 9]). Unfortunately, the argument that justifies such a uniqueness property cannot be adapted to general Hamiltonians (see [11, 15]).

Recent significant progress in the attempt to develop a more restrictive notion of singular characteristics is due to Khanin and Sobolevski [13]. In this paper, we will call such curves *strict singular characteristic* but in the literature they are also refereed to as *broken characteristics*, see [16, 17]. We now proceed to recall their definition: given a semiconcave solution *u* of ($\hbox {HJ}_{\mathrm{loc}}$), a Lipschitz singular curve $\mathbf {x}:[0,T]\rightarrow \Omega$ is called a strict singular characteristic from $x\in \text{ Sing }\,(u)$ if there exists a measurable selection $p(t)\in D^+u(\mathbf {x}(t))$ such that

1.3 $$\begin{aligned} \begin{aligned} {\left\{ \begin{array}{ll} \dot{\mathbf {x}}(t)=H_p(\mathbf {x}(t),p(t))&{} a.e.\ t\in [0,T],\\ \mathbf {x}(0)=x.&{} \end{array}\right. } \end{aligned} \end{aligned}$$

As already mentioned, the existence of strict singular characteristics (for a time dependent version of ($\hbox {HJ}_{\mathrm{loc}}$)) was proved in [13], where additional regularity properties of such curves were established, including the right-differentiability of $\mathbf {x}$ for every *t*, the right-continuity of $\dot{\mathbf {x}}$, and the fact that $p(\cdot ):[0,T]\rightarrow \mathbb {R}^n$ satisfies

1.4 $$\begin{aligned} H(\mathbf {x}(t),p(t))=\min _{p\in D^+u(\mathbf {x}(t))}H(\mathbf {x}(t),p)\quad \forall t\in [0,T]. \end{aligned}$$

In Appendix A, we give a proof of the existence and regularity of strict characteristics for solutions to ($\hbox {HJ}_{\mathrm{loc}}$) for the reader’s convenience.

In view of the above considerations, it is quite natural to raise the following questions:

1. What is the relation between a strict singular characteristic, $\mathbf {x}$, and a singular characteristic, $\mathbf {y}$, from the same initial point?
2. What kind of uniqueness result can be proved for singular characteristics? What about strict singular characteristics?

In this paper, we will answer the above questions in the two-dimensional case under the following additional conditions:

(A) $n=2$ and $\mathbf {y}$ is Lipschitz;
(B) the singular initial point $x_0=\mathbf {y}(0)$ of the singular characteristic $\mathbf {y}$ is not a critical point with respect the pair (*H*, *u*), i.e., $0\not \in H_p(x_0,D^+u(x_0))$;
(C) $\mathbf {y}$ is right differentiable at 0 and
  $$\begin{aligned} \dot{\mathbf {y}}^+(0)=H(x_0,p_0), \end{aligned}$$
  where $$p_0=\arg \min \{H(x_0,p): p\in D^+u(x_0)\}$$;
(D) $$\lim _{t\rightarrow 0^+}{\text {*}}{ess\ sup}_{s\in [0,t]}|\dot{\mathbf {y}}(s)-\dot{\mathbf {y}}^+(0)|=0$$.

The meaning of conditions (A) is clear. Condition (B) ensures the fact that singular characteristics are not constant. The right differentiability of singular characteristics at 0 and the essential right continuity of $\dot{\mathbf {y}}$ at 0 are crucial properties to our approach. On the one hand, together with condition (B) they ensure that a singular characteristic is a genuine arc near $t=0$. On the other hand, (D) is essential to construct the change of variable on which our uniqueness result is based. Notice that any strict singular characteristic $\mathbf {x}$ and the singular characteristic $\mathbf {y}$ given in [11] (see also Proposition 2.12) satisfy conditions (A)–(D) provided that the initial point is not critical. The intrinsic singular characteristic $\mathbf {z}$ constructed in [4] (see also Proposition 2.13) satisfies just conditions (A)–(C), in general.

The main results of this paper can be described as follows.

- For any pair of singular curves $\mathbf {x}_1$ and $\mathbf {x}_2$ satisfying condition (A)-(D), there exists $\tau >0$ and a bi-Lipschitz homeomorphism $\phi :[0,\tau ]\rightarrow [0,\phi (\tau )]$ such that, $\mathbf {x}_1(\phi (t))=\mathbf {x}_2(t)$ for all $t\in [0,\tau ]$. In other words, the singular characteristic staring from a non-critical point *x* is unique up to a bi-Lipschitz reparametrization (Theorem 3.6).
- In particular, if $\mathbf {x}$ is a strict singular characteristic and $\mathbf {y}$ is a singular characteristic starting from the same noncritical initial point *x*, then there exists $\tau >0$ and a bi-Lipschitz homeomorphism $\phi :[0,\tau ]\rightarrow [0,\phi (\tau )]$ such that $\mathbf {y}(\phi (t))=\mathbf {x}(t)$ for all $t\in [0,\tau ]$ (Corollary 3.8).
- We have the following *uniqueness* property for strict singular characteristics: let
  $$\begin{aligned} \mathbf {x}_j:[0,T]\rightarrow \Omega \quad (j=1,2) \end{aligned}$$
  be strict singular characteristics from the same noncritical initial point *x*. Then there exists $\tau \in (0, T]$ such that $\mathbf {x}_1(t)=\mathbf {x}_2(t)$ for all $t\in [0,\tau ]$. (Theorem 3.9)

Finally, we remark that the results of this paper cannot be applied to intrinsic singular characteristics because of the mentioned lack of condition (D). Extra techniques will have to be developed to cover such an important class.

The paper is organized as follows. In Sect. 2, we introduce necessary material on Hamilton–Jacobi equations, semiconcavity, and singular characteristics. In Sect. 3, we answer question (Q1)–(Q2) in the two-dimensional case. In the appendix, we give a detailed proof of the existence result for strict singular characteristics.

## Hamilton–Jacobi equation and semiconcavity

In this section, we review some basic facts on semiconcave functions and Hamilton–Jacobi equations.

### Semiconcave function

Let $\Omega \subset \mathbb {R}^n$ be a convex open set. We recall that a function $u:\Omega \rightarrow \mathbb {R}$ is *semiconcave* (with linear modulus) if there exists a constant $C>0$ such that

2.1 $$\begin{aligned} \lambda u(x)+(1-\lambda )u(y)-u(\lambda x+(1-\lambda )y)\leqslant \frac{C}{2}\lambda (1-\lambda )|x-y|^2 \end{aligned}$$

for any $x,y\in \Omega$ and $\lambda \in [0,1]$.

Let $u:\Omega \subset \mathbb {R}^n\rightarrow \mathbb {R}$ be a continuous function. For any $x\in \Omega$, the closed convex sets

$$\begin{aligned} D^-u(x)&=\left\{ p\in \mathbb {R}^n:\liminf _{y\rightarrow x}\frac{u(y)-u(x)-\langle p,y-x\rangle }{|y-x|}\geqslant 0\right\} ,\\ D^+u(x)&=\left\{ p\in \mathbb {R}^n:\limsup _{y\rightarrow x}\frac{u(y)-u(x)-\langle p,y-x\rangle }{|y-x|}\leqslant 0\right\} . \end{aligned}$$

are called the *subdifferential* and *superdifferential* of *u* at *x*, respectively.

The following characterization of semiconcavity (with linear modulus) for a continuous function comes from proximal analysis.

#### Proposition 2.1

Let $u:\Omega \rightarrow \mathbb {R}$ be a continuous function. If there exists a constant $C>0$ such that, for any $x\in \Omega$, there exists $p\in \mathbb {R}^n$ such that

2.2 $$\begin{aligned} u(y)\leqslant u(x)+\langle p,y-x\rangle +\frac{C}{2}|y-x|^2,\quad \forall y\in \Omega , \end{aligned}$$

then *u* is semiconcave with constant *C* and $p\in D^+u(x)$. Conversely, if *u* is semiconcave in $\Omega$ with constant *C*, then (2.2) holds for any $x\in \Omega$ and $p\in D^+u(x)$.

Let $u:\Omega \rightarrow \mathbb {R}$ be locally Lipschitz. We recall that a vector $p\in \mathbb {R}^n$ is called a *reachable* (or *limiting*) *gradient* of *u* at *x* if there exists a sequence $\{x_n\}\subset \Omega {\setminus }\{x\}$ such that *u* is differentiable at $x_k$ for each $k\in \mathbb {N}$, and

$$\begin{aligned} \lim _{k\rightarrow \infty }x_k=x\quad \text {and}\quad \lim _{k\rightarrow \infty }Du(x_k)=p. \end{aligned}$$

The set of all reachable gradients of *u* at *x* is denoted by $D^{*}u(x)$.

The following proposition concerns fundamental properties of semiconcave funtions and their gradients (see [10] for the proof).

#### Proposition 2.2

Let $u:\Omega \subset \mathbb {R}^n\rightarrow \mathbb {R}$ be a semiconcave function and let $x\in \Omega$. Then the following properties hold.

- $D^+u(x)$ is a nonempty compact convex set in $\mathbb {R}^n$ and $D^{*}u(x)\subset \partial D^+u(x)$, where $\partial D^+u(x)$ denotes the topological boundary of $D^+u(x)$.
- The set-valued function $x\rightsquigarrow D^+u(x)$ is upper semicontinuous.
- If $D^+u(x)$ is a singleton, then *u* is differentiable at *x*. Moreover, if $D^+u(x)$ is a singleton for every point in $\Omega$, then $u\in C^1(\Omega )$.
- $$D^+u(x)=\mathrm {co}\, D^{*}u(x)$$.
- If *u* is both semiconcave and semiconvex in $\Omega$, then $u\in C^{1,1}(\Omega )$.

#### Definition 2.3

Let $u:\Omega \rightarrow \mathbb {R}$ be a semiconcave function. $x\in \Omega$ is called a *singular point* of *u* if $D^+u(x)$ is not a singleton. The set of all singular points of *u* is denoted by $\text{ Sing }\,(u)$.

#### Definition 2.4

Let $k\in \{0,1,\dots ,n\}$ and let $C\subset \mathbb {R}^n$. *C* is called a *k*-*rectifiable* set if there exists a Lipschitz continuous function $f:\mathbb {R}^k\rightarrow \mathbb {R}^n$ such that $C\subset f(\mathbb {R}^k)$. *C* is called a *countably* *k*-*rectifiable* set if it is the union of a countable family of *k*-rectifiable sets.

Let us recall a result on the rectifiability of the singular set $\text{ Sing }\,(u)$ of a semiconcave function *u* in dimension two.

#### Proposition 2.5

[10] Let $\Omega \subset \mathbb {R}^2$ be an open domain, $u:\Omega \rightarrow \mathbb {R}$ be a semiconcave function, and set

$$\begin{aligned} \text{ Sing}_k(u)=\{x\in \text{ Sing }\,(u): \dim (D^+u(x))=k\},\quad k=0,1,2. \end{aligned}$$

Then $\text{ Sing}_k(u)$ is countably $(2-k)$-rectifiable for $k=0,1,2$. In particular, $\text{ Sing}_2(u)$ is countable.

### Aspects of weak KAM theory

For any $x,y\in \mathbb {R}^n$ and $t>0$, we denote by $\Gamma ^t_{x,y}$ the set of all absolutely continuous curves $\xi$ defined on [0, *t*] such that $\xi (0)=x$ and $\xi (t)=y$. Define

2.3 $$\begin{aligned} A_t(x,y)=\inf _{\xi \in \Gamma ^t_{x,y}}\int ^t_0L(\xi (s),\dot{\xi }(s))\ ds,\quad x,y\in \mathbb {R}^n,\ t>0. \end{aligned}$$

We call $A_t(x,y)$ the *fundamental solution* for the Hamilton–Jacobi equation

$$\begin{aligned} D_tu(t,x)+H(x_0,D_xu(t,x))=0,\quad t>0, x\in \mathbb {R}^n. \end{aligned}$$

By classical results (Tonelli’s theory), the infimum in (2.3) is a minimum. Each curve $\xi \in \Gamma ^t_{x,y}$ attaining such a minimum is called a *minimal curve for* $A_t(x,y)$.

#### Definition 2.6

For each $u:\mathbb {R}^n\rightarrow \mathbb {R}$, let and $T_tu$ and $\breve{T}_tu$ be the *Lax-Oleinik evolution of negative and positive type* defined, respectively, by

$$\begin{aligned} \begin{aligned} T_tu(x)=&\,\inf _{y\in \mathbb {R}^n}\{u(y)+A_t(y,x)\},\\ \breve{T}_tu(x)=&\,\sup _{y\in \mathbb {R}^n}\{u(y)-A_t(x,y)\}, \end{aligned} \quad (x\in \mathbb {R}^n, t>0). \end{aligned}$$

The following result is well-known.

#### Proposition 2.7

[12] There exists a Lipschitz semiconcave viscosity solution of ($\hbox {HJ}_s$). Moreover, such a solution *u* is a common fixed point of the semigroup $\{T_t\}$, i.e., $T_tu=u$ for all $t\geqslant 0$.

Clearly, ($\hbox {HJ}_s$) has no unique solution and we call each solution, given as a fixed point of the semigroup $\{T_t\}$, a *weak KAM solution* of ($\hbox {HJ}_s$).

#### Definition 2.8

Let *u* be a continuous function on *M*. We say *u* is *L*-*dominated* if

$$\begin{aligned} u(\xi (b))-u(\xi (a))\leqslant \int ^b_aL(\xi (s),\dot{\xi }(s))\ ds, \end{aligned}$$

for all absolutely continuous curves $\xi :[a,b]\rightarrow \mathbb {R}^n\;(a<b)$, with $\xi (a)=x$ and $\xi (b)=y$. We say such an absolutely continuous curve $\xi$ is a (*u*, *L*)-*calibrated curve*, or a *u*-*calibrated curve* for short, if the equality holds in the inequality above. A curve $\xi :(-\infty ,0]\rightarrow \mathbb {R}^n$ is called a *u*-calibrated curve if it is *u*-calibrated on each compact sub-interval of $(-\infty ,0]$. In this case, we also say that $\xi$ is a *backward calibrated curve* (with respect to *u*).

The following result explains the relation between the set of all reachable gradients and the set of all backward calibrated curves from *x* (see, e.g., [10] or [14] for the proof).

#### Proposition 2.9

Let $u:\mathbb {R}^n\rightarrow \mathbb {R}$ be a weak KAM solution of ($\hbox {HJ}_s$) and let $x\in \mathbb {R}^n$. Then $p\in D^{*}u(x)$ if and only if there exists a unique $C^2$ curve $\xi :(-\infty ,0]\rightarrow \mathbb {R}^n$ with $\xi (0)=x$ and $p=L_v(x,\dot{\xi }(0))$, which is a backward calibrated curve with respect to *u*.

### Propagation of singularities

In this paper, we will discuss various types of singular arcs describing the propagation of singularities for Lipschitz semiconcave solutions of the Hamilton–Jacobi equations ($\hbox {HJ}_{\mathrm{loc}}$) and ($\hbox {HJ}_s$).

#### Definition 2.10

$x_0$ is called a *critical point with respect to* (*H*, *u*) if $0\in H_p(x_0,D^+u(x))$.

Let *u* be a Lipschitz semiconcave viscosity solution of ($\hbox {HJ}_{\mathrm{loc}}$) and $x\in \text{ Sing }\,(u)$.

#### Definition 2.11

- A *singular characteristic from* $x_0$ is a Lipschitz arc $\mathbf {x}:[0,\tau ]{\rightarrow }\Omega (\tau {>}0)$ such that:
  1. $\mathbf {x}$ is a generalized characteristic with $\mathbf {x}(0)=x_0$,
  2. $$\mathbf {x}(t)\in \text{ Sing }\,(u)$$ for all $$t\in [0,\tau ]$$,
  3. $$\dot{\mathbf {x}}^+(0)=H_p(x_0,p_0)$$ where $$p_0=\arg \min \{H(x_0,p): p\in D^+u(x_0)\}$$,
  4. $$\lim _{t\rightarrow 0^+}{\text {*}}{ess\ sup}_{s\in [0,t]}|\dot{\mathbf {x}}(s)-\dot{\mathbf {x}}^+(0)|=0$$.
- A singular characteristic $\mathbf {x}:[0,T]\rightarrow \Omega$ from $x_0$ is called a *strict singular characteristic* if there exists a measurable selection $p(t)\in D^+u(\mathbf {x}(t))$ such that
  $$\begin{aligned} {\left\{ \begin{array}{ll} \dot{\mathbf {x}}(t)=H_p(\mathbf {x}(t),p(t))&{} a.e.\ t\in [0,T],\\ \mathbf {x}(0)=x_0.&{} \end{array}\right. } \end{aligned}$$

The following existence of singular characteristic is due to [1, 11].

#### Proposition 2.12

Let *u* be a Lipschitz semiconcave solution of ($\hbox {HJ}_{\mathrm{loc}}$) and $x\in \text{ Sing }\,(u)$. Then, there exists a singular characteristic $\mathbf {y}:[0,T]\rightarrow \Omega$ with $\mathbf {y}(0)=x$.

Now, suppose *u* is a Lipschitz semiconcave weak KAM solution of ($\hbox {HJ}_s$). In [4], another singular curve for *u* is constructed as follows. First, it is shown that there exists $\lambda _0>0$ such that for any $(t,x)\in \mathbb {R}_+\times \mathbb {R}^n$ and any maximizer *y* for the function $u(\cdot )-A_t(x,\cdot )$, we have that $|y-x|\leqslant \lambda _0 t$. Then, taking $\lambda =\lambda _0+1$, one shows that there exists $t_{0}>0$ such that, if $t\in (0,t_0]$, then there exists a unique $y_{t,x}\in B(x,\lambda t)$ of $u(\cdot )-A_t(x,\cdot )$ such that

2.4 $$\begin{aligned} \breve{T}_tu(x)=u(y_{t,x})-A_t(x,y_{t,x}). \end{aligned}$$

Moreover, such a $t_0$ is such that $-A_t(x,\cdot )$ is concave with constant $C_2/t$ and $C_1-C_2/t<0$ for $0<t\leqslant t_0$. We now define the curve

2.5 $$\begin{aligned} \mathbf {z}(t)= {\left\{ \begin{array}{ll} x,&{}t=0,\\ y_{t,x},&{} t\in (0,t_0]. \end{array}\right. } \end{aligned}$$

#### Proposition 2.13

[4] Let the curve $\mathbf {z}$ be defined in (2.5). Then, the following holds:

1. $$\mathbf {z}$$ is Lipschitz,
2. if $x\in \text{ Sing }\,(u)$ then $\mathbf {z}(t)\in \text{ Sing }\,(u)$ for all $t\in [0,t_0]$,
3. $$\dot{\mathbf {z}}^+(0)$$ exists and
  $$\begin{aligned} \dot{\mathbf {z}}^+(0)=H_p(x,p_0) \end{aligned}$$
  where $$p_0=\arg \min \{H(x,p): p\in D^+u(x)\}$$.

#### Definition 2.14

The Lipschitz arc $\mathbf {z}$ defined in (2.5) is called the *intrinsic characteristic* from $x\in \text{ Sing }\,(u)$.

## Singular characteristic on $\mathbb {R}^2$

We now return to questions (Q1) and (Q2) from the Introduction. So far, we have introduced three kinds of singular arcs issuing from a point $x_0\in \text{ Sing }\,(u)$, namely

- strict singular characteristics, that is, solutions to (1.3),
- singular characteristics, introduced in Definition 2.11, and
- the intrinsic singular characteristic $\mathbf {z}$ given by Proposition 2.13.

In this section, we will compare the first two notions of characteristics when $\Omega \subset \mathbb {R}^2$.

We begin by introducing the following class of Lipschitz arcs.

### Definition 3.1

Given $T>0$, we denote by $\text{ Lip}_0(0,T;\Omega )$ the class of all Lipschitz arcs ${\mathbf {x}}:[0,T]\rightarrow \Omega$ such that the right derivative

$$\begin{aligned} \dot{\mathbf {x}}^+(0)=\lim _{t\downarrow 0}\frac{\mathbf {x}(t)-\mathbf {x}(0)}{t} \end{aligned}$$

does exist and satisfies

3.1 $$\begin{aligned} \lim _{t\rightarrow 0^+}{\text {*}}{ess\ sup}_{s\in [0,t]}|\dot{\mathbf {x}}(s)-\dot{\mathbf {x}}^+(0)|=0. \end{aligned}$$

For any $\mathbf {x}\in \text{ Lip}_0(0,T;\Omega )$ we set

3.2 $$\begin{aligned} \omega _{\mathbf {x}}(t):={\text {*}}{ess\ sup}_{s\in [0,t]}|\dot{\mathbf {x}}(s)-\dot{\mathbf {x}}^+(0)|. \end{aligned}$$

Owing to (3.1), we have that $\omega _{\mathbf {x}}(t)\rightarrow 0$ as $t\downarrow 0$.

### Lemma 3.2

Let $\mathbf {x}\in \text{ Lip}_0(0,T;\Omega )$ be such that $\dot{\mathbf {x}}^+(0)\ne 0$. Then,

3.3 $$\begin{aligned} \big | |\mathbf {x}(t_1)-\mathbf {x}(t_0)|-|t_1-t_0|\cdot |\dot{\mathbf {x}}^+(0)|\big | \leqslant |t_1-t_0|\omega _{\mathbf {x}}(t_1\vee t_0)\quad \forall t_0,t_1\in [0,T] \end{aligned}$$

and $\mathbf {x}$ is injective on some interval $[0,T_0]$ with $0<T_0<T$.

### Proof

Observe that, for any $0\leqslant t_0\leqslant t_1\leqslant T$, the identity

$$\begin{aligned} \mathbf {x}(t_1)-\mathbf {x}(t_0)=\int ^{t_1}_{t_0}\dot{\mathbf {x}}(t)\ dt=(t_1-t_0)\dot{\mathbf {x}}^+(0)+\int ^{t_1}_{t_0}(\dot{\mathbf {x}}(t)-\dot{\mathbf {x}}^+(0))\ dt \end{aligned}$$

immediately gives (3.3). In turn, (3.3) implies that, if $\mathbf {x}(t_1)-\mathbf {x}(t_0)=0$, then

$$\begin{aligned} |t_1-t_0|\cdot |\dot{\mathbf {x}}^+(0)|\leqslant |t_1-t_0|\omega _{\mathbf {x}}(t_1) \end{aligned}$$

Since $\dot{\mathbf {x}}^+(0)\not =0$, we conclude that $t_1=t_0$ if $t_0, t_1\in [0, T_0]$ with $T_0$ sufficiently small.

Let $x\in \mathbb {R}^2$ and let $\theta \in \mathbb {R}^2$ be a unit vector. For any $\rho \in (0,1)$ let us consider the cone

3.4 $$\begin{aligned} C_\rho (x,\theta )=\big \{y\in \mathbb {R}^2~\big |~|\langle y-x,\theta \rangle |\geqslant \rho |y-x| \big \} \end{aligned}$$

with vertex in *x*, amplitude $\rho$, and axis $\theta$. Clearly, $C_\rho (x,\theta )$ is given by the union of the two cones

$$\begin{aligned} C^+_\rho (x,\theta )=\big \{y\in \mathbb {R}^2~\big |~\langle y-x,\theta \rangle \geqslant \rho |y-x| \big \} \end{aligned}$$

and

$$\begin{aligned} C^-_\rho (x,\theta )=\big \{y\in \mathbb {R}^2~\big |~\langle y-x,\theta \rangle \leqslant -\rho |y-x| \big \}, \end{aligned}$$

which intersect each other only at *x*.

### Lemma 3.3

Let $\mathbf {x}_j\in \text{ Lip}_0(0,T;\Omega )$ ($j=1,2$) be such that

(i) $$\mathbf {x}_1(0)= \mathbf {x}_2(0)=:x_0$$,
(ii) $$\dot{\mathbf {x}}_1^+(0)=\dot{\mathbf {x}}_2^+(0)$$, and
(iii) $$\dot{\mathbf {x}}_j(s)\ne 0$$ ($$j=1,2$$) for a.e. $$s\in [0,T]$$.

Define

3.5 $$\begin{aligned} \theta _1(s)= \frac{\dot{\mathbf {x}}_1(t)}{|\dot{\mathbf {x}}_1(t)|}\quad (s\in [0,T] \text{ a.e.}) \end{aligned}$$

and fix $\rho \in (0,1)$. Then the following holds true:

- there exists $s_\rho \in (0,T]$ such that $x_0\in C^-_\rho (\mathbf {x}_1(s),\theta _1(s))$ for a.e. $s\in [0,s_\rho ]$;
- there exists $\tau _\rho \in (0,T]$ such that for all $t\in (0,\tau _\rho ]$ there exists $\sigma _\rho (t)\in (0,T]$ such that
  3.6 $$\begin{aligned}&|\mathbf {x}_2(t)-\mathbf {x}_1(s)|\leqslant \frac{1+\rho }{2\rho } t|\dot{\mathbf {x}}_1^+(0)|\quad \forall s\in [0,\sigma _\rho (t)] \end{aligned}$$
  3.7 $$\begin{aligned}&\mathbf {x}_2(t)\in C^+_\rho (\mathbf {x}_1(s),\theta _1(s))\quad \text{ for } \text{ a.e. } s\in [0,\sigma _\rho (t)]. \end{aligned}$$

### Proof

Hereafter, we denote by $o_i(s)\,(i\in \mathbb {N})$ any (scalar- or vector-valued) function such that

$$\begin{aligned} \lim _{s\rightarrow 0^+}\frac{o_i(s)}{s}=0. \end{aligned}$$

In view of (3.3) we conclude that

3.8 $$\begin{aligned} |x_0-\mathbf {x}_1(s)|=s|\dot{\mathbf {x}}_1^+(0)|+o_1(s)\quad \forall s\in [0,T]. \end{aligned}$$

Moreover, setting $\theta _1(0)=\dot{\mathbf {x}}_1^+(0)/|\dot{\mathbf {x}}_1^+(0)|$, for a.e. $s\in [0,T]$ we have that

3.9 $$\begin{aligned} \begin{aligned} \langle x_0-\mathbf {x}_1(s),\theta _1(s)\rangle =&\,\langle x_0-\mathbf {x}_1(s),\theta _1(0)\rangle +\langle x_0-\mathbf {x}_1(s),\theta _1(s)-\theta _1(0)\rangle \\ =&\,-s|\dot{\mathbf {x}}^+_1(0)|+o_2(s). \end{aligned} \end{aligned}$$

Now, having fixed $\rho \in (0,1)$ let $s_\rho \in (0,T_1]$ be such that, for a.e. $s\in [0,s_\rho ]$,

$$\begin{aligned} \frac{|o_1(s)|}{s} \leqslant \frac{1-\rho }{2\rho } |\dot{\mathbf {x}}^+_1(0)|\quad \text{ and }\quad \frac{|o_2(s)|}{s} \leqslant \frac{1-\rho }{2} |\dot{\mathbf {x}}^+_1(0)|. \end{aligned}$$

Then $|x_0-\mathbf {x}_1(s)|\leqslant \frac{1+\rho }{2\rho }s|\dot{\mathbf {x}}_1^+(0)|$ by (3.8). From (3.9) it follows that

$$\begin{aligned} \langle x_0-\mathbf {x}_1(s),\theta _1(s)\rangle \leqslant -\frac{1+\rho }{2} s |\dot{\mathbf {x}}^+_1(0)| \leqslant -\rho |x_0-\mathbf {x}_1(s)| \quad (s\in [0,s_\rho ] \text{ a.e.}) \end{aligned}$$

and (a) follows.

The proof of (b) is similar: since $\dot{\mathbf {x}}_2^+(0)=\dot{\mathbf {x}}_1^+(0)$ by condition (ii), for all $t\in [0,T]$ and $s\in [0,T]$ we have that

3.10 $$\begin{aligned} \mathbf {x}_2(t)-\mathbf {x}_1(s)=(t-s)\dot{\mathbf {x}}_1^+(0) +o_3(t)+o_3(s). \end{aligned}$$

Hence, for all $s,t\in (0,T]$ we deduce that

$$\begin{aligned} \Big |\frac{\mathbf {x}_2(t)-\mathbf {x}_1(s)}{t|\dot{\mathbf {x}}_1^+(0)|}- \frac{\dot{\mathbf {x}}^+_1(0)}{|\dot{\mathbf {x}}^+_1(0)|}\Big |\leqslant \, \frac{s}{t}+\frac{|o_3(t)|+|o_3(s)|}{t|\dot{\mathbf {x}}_1^+(0) |}. \end{aligned}$$

So,

3.11 $$\begin{aligned} |\mathbf {x}_2(t)-\mathbf {x}_1(s)|\leqslant t|\dot{\mathbf {x}}_1^+(0)| \Big ( 1+ \frac{s}{t}+\frac{|o_3(t)|+|o_3(s)|}{t|\dot{\mathbf {x}}_1^+(0) |}\Big ). \end{aligned}$$

Next, take the scalar product of each side of (3.10) with $\theta _1(s)$ to obtain

3.12 $$\begin{aligned}&\langle \mathbf {x}_2(t)-\mathbf {x}_1(s),\theta _1(s)\rangle =t\langle \dot{\mathbf {x}}_1^+(0),\theta _1(s)\rangle -\langle s\dot{\mathbf {x}}_1^+(0)-o_3(t)-o_3(s),\theta _1(s)\rangle \nonumber \\&\quad =t|\dot{\mathbf {x}}_1^+(0)|+t \langle \dot{\mathbf {x}}_1^+(0),\theta _1(s)-\theta _1(0)\rangle -\langle s\dot{\mathbf {x}}_1^+(0)-o_3(t)-o_3(s),\theta _1(s)\rangle \end{aligned}$$

for all $t\in [0,T]$ and a.e. $s\in [0,T]$.

Once again, having fixed $\rho \in (0,1)$, we can find $\tau _\rho \in (0,T]$ satisfying the following: for all $t\in (0,\tau _\rho ]$ there exists $\sigma _\rho (t)\in (0,T]$ such that

$$\begin{aligned} t| \langle \dot{\mathbf {x}}_1^+(0),\theta _1(s)-\theta _1(0)\rangle |+ |\langle s\dot{\mathbf {x}}_1^+(0)-o_3(t)-o_3(s),\theta _1(s)\rangle | \leqslant \frac{1-\rho }{2} t|\dot{\mathbf {x}}_1^+(0)| \end{aligned}$$

and

$$\begin{aligned} 1+ \frac{s}{t}+\frac{|o_3(t)|+|o_3(s)|}{t|\dot{\mathbf {x}}_1^+(0) |}\leqslant \frac{1+\rho }{2\rho } \end{aligned}$$

for all $t\in [0,\tau _\rho ]$ and a.e. $s\in [0,\sigma _\rho (t)]$. Then, (3.11) leads directly to (3.6). Moreover, returning to (3.12), for all $t\in [0,\tau _\rho ]$ and a.e. $s\in [0,\sigma _\rho (t)]$ we conclude that

$$\begin{aligned} \langle \mathbf {x}_2(t)-\mathbf {x}_1(s),\theta _1(s)\rangle \geqslant t|\dot{\mathbf {x}}_1^+(0)|-\frac{1-\rho }{2} t|\dot{\mathbf {x}}_1^+(0)| =\frac{1+\rho }{2} t|\dot{\mathbf {x}}_1^+(0)| \geqslant \rho |\mathbf {x}_2(t)-\mathbf {x}_1(s)|, \end{aligned}$$

where we have used (3.11) to deduce the last inequality. Hence, (3.7) follows.

Given a semiconcave solution *u* of ($\hbox {HJ}_{\mathrm{loc}}$), we hereafter concentrate on singular arcs for *u*, that is, arcs $\mathbf {x}\in \text{ Lip}_0(0,T;\Omega )$ such that $\mathbf {x}(t)\in \text{ Sing }\,(u)$ for all $t\in [0,T]$. We denote such a subset of $\text{ Lip}_0(0,T;\Omega )$ by $\text{ Lip}^u_0(0,T;\Omega )$.

### Lemma 3.4

Let *u* be a semiconcave solution of ($\hbox {HJ}_{\mathrm{loc}}$) and let $\mathbf {x}\in \text{ Lip}^u_0(0,T;\Omega )$ be such that $\dot{\mathbf {x}}^+(0)\ne 0$. Then there exists $T_0\in (0,T]$ such that the set

$$\begin{aligned} S_{\mathbf {x}}=\Big \{s\in [0,T_0]~\big |~D^+u(\mathbf {x}(s))=[p_s^1,p_s^2]\;\text{ with }\; p_s^1, p_s^2\in D^*u(\mathbf {x}(s))\,,\;p_s^1\ne p_s^2\Big \}. \end{aligned}$$

has full measure in $[0,T_0]$. Moreover, $\lim _{s\rightarrow 0^+}p_s^i=p_0^i$ with $p_0^i\in D^*u(x_0)\;(i=1,2)$ and

3.13 $$\begin{aligned} \langle \dot{\mathbf {x}}(s),p_s^2-p_s^1\rangle =0\quad \text{ for } \text{ a.e. } s\in [0,T_0] \end{aligned}$$

### Proof

The structure of the superdifferential of *u* along $\mathbf {x}$ is described by Proposition 2.5 and Proposition 3.3.15 in [10].

### Lemma 3.5

Let *u* be a semiconcave solution of ($\hbox {HJ}_{\mathrm{loc}}$) and let $x_0\in \text{ Sing }\,(u)$ be such that $0\not \in H_p(x_0,D^+u(x_0))$. Let $\mathbf {x}\in \text{ Lip}_0^u(0,T;\Omega )$ be such that $\mathbf {x}(0)=x_0$ and

$$\begin{aligned} \dot{\mathbf {x}}^+(0)=H_p(x_0,p_0)\quad \text {where}\ p_0=\arg \min \{H(x_0,p): p\in D^+u(x_0)\}. \end{aligned}$$

Let $T_0\in (0,T]$ be given by Lemma 3.4 and, for every $s\in S_\mathbf {x}$, let $\xi ^1_s$ and $\xi ^2_s$ be backward calibrated curves on $(-\infty ,0]$ satisfying

3.14 $$\begin{aligned} \xi _s^i(0)=\mathbf {x}(s)\quad \text{ and } \quad \dot{\xi }_s^i(0)=H_p(\mathbf {x}(s), p^i_{s}) \quad (i=1,2) \end{aligned}$$

Then there exist constants $r_1>0$, $s_1\in (0,T_0]$, and $\delta \in (0,1)$ and such that

3.15 $$\begin{aligned} |\mathbf {x}(s)-\xi ^i_s(-r)|\geqslant \delta r \quad (i=1,2) \end{aligned}$$

and, for all $s\in [0,s_1]\cap S_\mathbf {x}$ and $r\in [0,r_1]$,

3.16 $$\begin{aligned} \xi ^1_s(-r)\in C^+_\delta (\mathbf {x}(s),\theta _2(s))\quad \text{ and }\quad \xi ^2_s(-r)\in C^-_\delta (\mathbf {x}(s),\theta _2(s)) \end{aligned}$$

where

$$\begin{aligned} \theta _2(s)=\frac{p^2_s-p^1_s}{|p^2_s-p^1_s|}\quad (s\in S_\mathbf {x}). \end{aligned}$$

### Proof

The existence of backward calibrated curves satisfying (3.14) follows from Proposition 2.9. Moreover, for all $r\geqslant 0$ we have that

3.17 $$\begin{aligned} \mathbf {x}(s)-\xi ^i_s(-r)=\xi ^i_s(0)-\xi ^i_s(-r)=r\dot{\xi }^i(0)+o(r)= rH_p(\mathbf {x}(s), p^i_{s})+o(r) \quad (i=1,2) \end{aligned}$$

where $\lim _{r\rightarrow 0^+}o(r)/r=0$ uniformly with respect to $s\in S_\mathbf {x}$.

Now, observe that, since $x_0$ is not a critical point with respect to (*u*, *H*), by possibly reducing $T_0$ we have that $\mathbf {x}(s)$ is also not a critical point for all $s\in [0,T_0]$ due to the upper-semicontinuity of the set-valued map $s\rightrightarrows H_p(\mathbf {x}(s),D^+u(\mathbf {x}(s)))$. So, for some $r_0>0$, $s_0\in (0,T_1]$, and $\delta _0\in (0,1)$, we deduce that

3.18 $$\begin{aligned} \frac{r}{\delta _0}\geqslant |\mathbf {x}(s)-\xi ^i_s(-r)|=r|H_p(\mathbf {x}(s), p^i_{s})|+o(r)\geqslant \delta _0 r\quad (i=1,2) \end{aligned}$$

for all $s\in [0,s_0]\cap S_\mathbf {x}$ and $r\in [0,r_0]$. This proves (3.15).

Next, recall that $H(x_0,p^i_0)=0$ because $p_0^i\in D^*u(x_0)\;(i=1,2)$. So, by the strict convexity of $H(x_0,\cdot )$, we deduce that there exists $\nu >0$ such that

$$\begin{aligned} \langle H_p(x_0,p^2_0),p^2_0-p^1_0\rangle \geqslant&\,H(x_0,p^2_0)-H(x_0,p^1_0)+\nu |p^2_0-p^1_0|^2=\nu |p^2_0-p^1_0|^2>0\\ \langle H_p(x_0,p^1_0),p^2_0-p^1_0\rangle \leqslant&\,H(x_0,p^2_0)-H(x_0,p^1_0)-\nu |p^2_0-p^1_0|^2=-\nu |p^2_0-p^1_0|^2<0 \end{aligned}$$

Hence, the upper-semicontinuity of the set-valued map $s\rightrightarrows H_p(\mathbf {x}(s),D^+u(\mathbf {x}(s)))$ ensures the existence of numbers $\delta _1\in (0,1)$ and $s_1\in (0,s_0]$ such that

3.19 $$\begin{aligned} \langle H_p(\mathbf {x}(s),p^2_s),\theta _2(s)\rangle \geqslant \delta _1,\quad \langle H_p(\mathbf {x}(s),p^1_s),\theta _2(s)\rangle \leqslant -\delta _1 \quad \forall s\in [0,s_1]\cap S. \end{aligned}$$

Therefore, combining (3.17) and (3.19), we conclude that, after possibly replacing $r_0$ by a smaller nummber $r_1>0$,

$$\begin{aligned} \langle \xi _s(-r)-\mathbf {x}(s),\theta _2(s)\rangle =-r\langle H_p(\mathbf {x}(s),p^1_s),\theta _2(s)\rangle +o(r) \geqslant r\delta _1+o(r)\geqslant r\frac{\delta _1}{2} \end{aligned}$$

for all $s\in [0,s_1]\cap S_\mathbf {x}$ and $r\in [0,r_1]$. By (3.18) and the above inequality we have that $\xi ^1_s(-r)\in C^+_\delta (\mathbf {x}(s),\theta _2(s))$ with $\delta = \delta _0\delta _1/2$.

The analogous statement for $\xi ^2_s$ in (3.16) can be proved by a similar argument.

We are now ready to state our main result, which ensures that singular curves coincide up to a bi-Lipschitz reparameterization, at least when *x* is not a critical point.

### Theorem 3.6

Let *u* be a semiconcave solution of ($\hbox {HJ}_{\mathrm{loc}}$) and let $x_0\in \text{ Sing }\,(u)$ be such that $0\not \in H_p(x_0,D^+u(x_0))$. Let $\mathbf {x}_j\in \text{ Lip}_0^u(0,T;\Omega )$ ($j=1,2$) be such that $\mathbf {x}_j(0)=x_0$ and

$$\begin{aligned} \dot{\mathbf {x}}_j^+(0)=H_p(x_0,p_0) \text{ where } p_0=\arg \min \{H(x_0,p): p\in D^+u(x_0)\}. \end{aligned}$$

Then, there exists $\sigma \in (0,T]$ such that there exists a unique bi-Lipschitz homeomorphism

$$\phi :[0,\sigma ]\rightarrow [0,\phi (\sigma )]\subset [0,T_2]$$

satisfying $\mathbf {x}_1(s)=\mathbf {x}_2(\phi (s))$ for all $s\in [0,\sigma ]$.

We begin the proof with the following lemma.

### Lemma 3.7

Under all assumptions of Theorem 3.6, there exists $\sigma \in (0,T]$ such that for all $s\in [0,\sigma ]$ there exists a unique $t_s\in [0,T]$ satisfying $\mathbf {x}_2(t_s)=\mathbf {x}_1(s)$.

### Proof

First, reduce $T>0$ in order to ensure that $\mathbf {x}_1$ and $\mathbf {x}_2$ are both injective on [0, *T*] and satisfy $\dot{\mathbf {x}}_j(s)\ne 0$ for a.e. $s\in [0,T]$ ($j=1,2$).

Then, observe that Lemma 3.5, applied to $\mathbf {x}=\mathbf {x}_1$, ensures the existence of $r_1>0$, $s_1\in (0,T]$, and $\delta \in (0,1)$ such that for a.e. $s\in [0,s_1]$ one can find backward calibrated curves $\xi ^1_s$ and $\xi ^2_s$ on $(-\infty ,0]$ satisfying (3.14), (3.15), and (3.16) for all $r\in [0,r_1]$.

Next, choose

$$\begin{aligned} \rho =\frac{1+\sqrt{1-\delta ^2}}{2}\in \big ( \sqrt{1-\delta ^2},1\big ) \end{aligned}$$

in Lemma 3.3 and let $s_\rho$, $\tau _\rho$, and $\sigma _\rho (\cdot )$ be such that

(i) $$x_0\in C^-_\rho (\mathbf {x}_1(s),\theta _1(s))$$ for a.e. $$s\in [0,s_\rho ]$$,
(ii) $\mathbf {x}_2(t)\in C^+_\rho (\mathbf {x}_1(s),\theta _1(s))$ for all $t\in [0,\tau _r]$ and a.e. $s\in [0,\sigma _\rho (t)]$,
(iii) $|\mathbf {x}_2(t)-\mathbf {x}_1(s)|\leqslant \frac{1+\rho }{2\rho } t|\dot{\mathbf {x}}_1^+(0)|$ for all $t\in [0,\tau _r]$ and all $s\in [0,\sigma _\rho (t)]$.

**Fig. 1:**
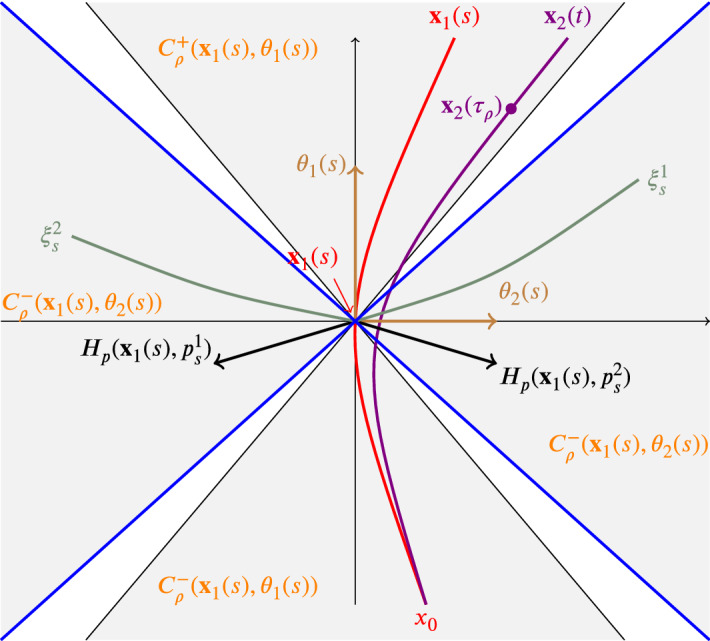
The illustration of various objects near $\mathbf {x}_1(s)$ for sufficiently small $s>0$

By possibly reducing $\tau _\rho$, without loss of generality we can suppose that (Fig. 1)

3.20 $$\begin{aligned} \frac{1+\rho }{2\rho } \tau _\rho |\dot{\mathbf {x}}_1^+(0)|<\delta r_1. \end{aligned}$$

Then, recalling that

$$\begin{aligned} \theta _1(s)= \frac{\dot{\mathbf {x}}_1(s)}{|\dot{\mathbf {x}}_1(s)|} \quad \text{ and }\quad \theta _2(s)=\frac{p^2_s-p^1_s}{|p^2_s-p^1_s|}\quad (s\in [0,T] \text{ a.e.}) \end{aligned}$$

are orthogonal unit vectors, we claim that, for a.e. $0\leqslant s\leqslant s_1\wedge \sigma _\rho (\tau _\rho )$,

$$\begin{aligned} C_\rho (\mathbf {x}_1(s),\theta _1(s))\bigcap C_\delta (\mathbf {x}_1(s),\theta _2(s))=\{\mathbf {x}_1(s)\}. \end{aligned}$$

Indeed, for any $x\in C_\rho (\mathbf {x}_1(s),\theta _1(s))\cap C_\delta (\mathbf {x}_1(s),\theta _2(s))$ we have that

$$\begin{aligned} |x-\mathbf {x}_1(s)|^2=&\,\langle x-\mathbf {x}_1(s),\theta _1(s)\rangle ^2+\langle x-\mathbf {x}_1(s),\theta _2(s)\rangle ^2\\ \geqslant&\,(\rho ^2+\delta ^2)|x-\mathbf {x}_1(s)|^2. \end{aligned}$$

This yields $x=\mathbf {x}_1(s)$ because $\rho ^2+\delta ^2>1$.

Now, define $\sigma =\min \big \{s_1, s_\rho , \sigma _\rho (\tau _\rho )\big \}$ and fix $s\in [0,\sigma ]$ in the set of full measure on which (i) is satisfied together with (ii) and (iii), that is,

$$\begin{aligned} \mathbf {x}_2(\tau _\rho )\in C^+_\rho (\mathbf {x}_1( s),\theta _1( s)) \quad \text{ and }\quad |\mathbf {x}_2(\tau _\rho )-\mathbf {x}_1( s)|<\delta r_1 \end{aligned}$$

where (3.20) has also been taken into account. By possibly reducing $\sigma$, we also have that $|\mathbf {x}_2(t)-\mathbf {x}_1( s)|<\delta r_1$ for all $t\in [0,\tau _\rho ]$. So, the arc $\mathbf {x}_2$, restricted to $[0,\tau _\rho ]$, connects the point $\mathbf {x}_2(\tau _\rho )$ of the cone $C^+_\rho (\mathbf {x}_1( s),\theta _1( s))$ with $x_0\in C^-_\rho (\mathbf {x}_1( s),\theta _1( s))$, remaining in the open ball of radius $\delta r_1$ centered at $\mathbf {x}_1( s)$. Thus, in view of (3.15) and (3.16), $\mathbf {x}_2$ must intersect at least one of the two calibrated curves $\xi ^1_s$ and $\xi ^2_s$.^1^ However, this can happen only at $\xi ^1_s(0)=\mathbf {x}_1( s)=\xi ^2_s(0)$, because *u* is smooth at all points $\xi ^2_s(-r)$ with $0<r<\infty$, whereas $\mathbf {x}_2$ is a singular arc. Finally, such an intersection occurs at a unique time $t_s$ owing to Lemma 3.2.

To complete the proof we observe that $\mathbf {x}_2(t_s)=\mathbf {x}_1(s)$ for all $s\in [0,\sigma ]$, not just on a set of full measure. This fact can be easily justified by an approximation argument.

We are now in a position to prove our main result.

### Proof of Theorem 3.6

Let $\sigma \in (0,T]$ be given by Lemma 3.7. Then for each $s\in [0,\sigma ]$ there exists a unique $\phi (s):=t_s\in [0,T_1]$ with $\mathbf {x}_2(\phi (s))=\mathbf {x}_1(s)$.

Recalling that, thanks to Lemma 3.2, both $\mathbf {x}_1(\cdot )$ and $\mathbf {x}_2(\cdot )$ can be assumed to be injective on $[0,\sigma ]$ and $[0,\phi (\sigma )]$, respectively, we proceed to show that $\phi$ is also an injection. Observe that, for any $0\leqslant s_0, s_1\leqslant \sigma$,

$$\begin{aligned} \mathbf {x}_2(\phi (s_1))-\mathbf {x}_2(\phi (s_0))=&\,\int ^{\phi (s_1)}_{\phi (s_0)}\dot{\mathbf {x}}_2(t)\ dt\\ =&\,\int ^{\phi (s_1)}_{\phi (s_0)}(\dot{\mathbf {x}}_2(t)-\dot{\mathbf {x}}_2^+(0))\ dt+(\phi (s_1)-\phi (s_0))\dot{\mathbf {x}}_2^+(0). \end{aligned}$$

Therefore,

$$\begin{aligned} |\mathbf {x}_2(\phi (s_1))-\mathbf {x}_2(\phi (s_0))-(\phi (s_1)-\phi (s_0))\dot{\mathbf {x}}_2^+(0)|\leqslant \omega _{\mathbf {x}_2}(\phi (s_1)\vee \phi (s_0))|\phi (s_1)-\phi (s_0)|, \end{aligned}$$

where $\omega _{\mathbf {x}_2}$ is given by (3.2). Thus, returning to $\mathbf {x}_1=\mathbf {x}_2\circ \phi$ we derive

3.21 $$\begin{aligned} \begin{aligned} |\mathbf {x}_1(s_1)-\mathbf {x}_1(s_0)|\geqslant&\,|\phi (s_1)-\phi (s_0)|\big (|\dot{\mathbf {x}}_2^+(0)|-\omega _{\mathbf {x}_2}(\phi (s_1)\vee \phi (s_0))|\big ),\\ |\mathbf {x}_1(s_1)-\mathbf {x}_1(s_0)|\leqslant&\,|\phi (s_1)-\phi (s_0)|\big (|\dot{\mathbf {x}}_2^+(0)|+\omega _{\mathbf {x}_2}(\phi (s_1)\vee \phi (s_0))|\big ). \end{aligned} \end{aligned}$$

Notice that (3.21) leads to

3.22 $$\begin{aligned} |\phi (s_1)-\phi (s_0)|\geqslant \frac{|\mathbf {x}_1(s_1)-\mathbf {x}_1(s_0)|}{|\dot{\mathbf {x}}_2^+(0)|+\omega _{\mathbf {x}_2}(\phi (s_1)\vee \phi (s_0))|} \end{aligned}$$

and this implies that $\phi$ is injective as so is $\mathbf {x}_1$.

Next, we prove that $\phi$ is continuous on $[0,\sigma ]$, or the graph of $\phi$ is closed. Let $s_j\rightarrow \bar{s}$ be any sequence such that $\phi (s_j)\rightarrow \bar{t}$ as $j\rightarrow \infty$. Then

$$\begin{aligned} \mathbf {x}_1(s_j)\rightarrow \mathbf {x}_1(\bar{s}) \quad \text{ and }\quad \mathbf {x}_2(\phi (s_j))=\mathbf {x}_1(s_j)\rightarrow \mathbf {x}_2(\bar{t}) \quad \text{ as }\quad j\rightarrow \infty . \end{aligned}$$

So, $\mathbf {x}_2(\phi (\bar{s}))=\mathbf {x}_1(\bar{s})=\mathbf {x}_2(\bar{t})$. Since $\mathbf {x}_2(\cdot )$ is injective, it follows that $\bar{t}=\phi (\bar{s})$.

Being continuous, $\phi$ is a homeomorphism. It remains to prove that $\phi$ is bi-Lipschitz. The continuity of $\phi$ at 0 ensures that, after possibly reducing $\sigma$,

3.23 $$\begin{aligned} \omega _{\mathbf {x}_1}(\phi (s_1)),\,\omega _{\mathbf {x}_2}(\phi (s_2))\leqslant \frac{|\dot{\mathbf {x}}_2^+(0)|}{2}=\frac{|\dot{\mathbf {x}}_1^+(0)|}{2} \end{aligned}$$

for all $s_0,s_1\in [0,\sigma ]$. Thus, by (3.21) we have that

$$\begin{aligned} |\phi (s_1)-\phi (s_0)|\leqslant \frac{|\mathbf {x}_1(s_1)-\mathbf {x}_1(s_0)|}{|\dot{\mathbf {x}}_2^+(0)|-\omega _{\mathbf {x}_2}(\phi (s_1)\vee \phi (s_0))|} \leqslant \frac{2\,\text{ Lip }\,(\mathbf {x}_1)}{|\dot{\mathbf {x}}_1^+(0)|}\cdot |s_1-s_0| \end{aligned}$$

for all $s\in [0,\sigma ]$ and $t\in [0,\phi (\sigma )]$. So, $\phi$ is Lipschitz on $[0,\sigma ]$. The fact that $\phi ^{-1}$ is also Lipschitz follows by a similar argument. Indeed, writing (3.22) for $t_i=\phi (s_i)$ and appealing to Lemma 3.2 and (3.23) once again we obtain

$$\begin{aligned} |t_1-t_0|\geqslant & {} \frac{|\mathbf {x}_2(t_1)-\mathbf {x}_2(t_0)|}{|\dot{\mathbf {x}}_2^+(0)|+\omega _{\mathbf {x}_2}(t_1\vee t_0)}=\frac{|\mathbf {x}_1(s_1)-\mathbf {x}_1(s_0)|}{|\dot{\mathbf {x}}_2^+(0)|+\omega _{\mathbf {x}_2}(t_1\vee t_0)} \\\geqslant & {} \frac{|\dot{\mathbf {x}}_1^+(0)|-\omega _{\mathbf {x}_1}(s_1\vee s_0)}{|\dot{\mathbf {x}}_2^+(0)|+\omega _{\mathbf {x}_2}(t_1\vee t_0)}\cdot |s_1-s_0| \geqslant \frac{1}{3} \cdot |s_1-s_0| \end{aligned}$$

The proof is completed noting that $\phi$ is unique due to the injectivity of $\mathbf {x}_1$ and $\mathbf {x}_2$.

### Corollary 3.8

Let $\mathbf {x}$ be a strict singular characteristic as in (1.3) and let $\mathbf {y}$ be any singular characteristic as in Proposition 2.12. If $x_0$ is not a critical point with respect to (*H*, *u*), then there exists $\sigma >0$ and a bi-Lipschitz homeomorphism $\phi :[0,\sigma ]\rightarrow [0,\phi (\sigma )]$ such that $\mathbf {y}(\phi (s))=\mathbf {x}(s)$ for all $s\in [0,\sigma ]$.

For strict singular characteristics, uniqueness holds without reparameterization as we show next.

### Theorem 3.9

Let *u* be a semiconcave solution of ($\hbox {HJ}_{\mathrm{loc}}$) and let $x_0\in \text{ Sing }\,(u)$ be such that $0\not \in H_p(x_0,D^+u(x_0))$. Let $\mathbf {x}_j:[0,T]\rightarrow \Omega$ ($j=1,2$) be strict singular characteristics with initial point $x_0$. Then there exists $\tau \in (0, T]$ such that $\mathbf {x}_1(t)=\mathbf {x}_2(t)$ for all $t\in [0,\tau ]$.

### Proof

By Theorem 3.6 there exists a bi-Lipschitz homeomorphism $\phi :[0,\tau _1]\rightarrow [0,\tau _2]$, with $0\leqslant \tau _j\leqslant T\,(j=1,2)$, such that

3.24 $$\begin{aligned} \mathbf {x}_1(t)=\mathbf {x}_2(\phi (t))\quad \forall t\in [0,\tau _1]. \end{aligned}$$

Moreover, since $\mathbf {x}_1$ and $\mathbf {x}_2$ are strict characteristics we have that

$$\begin{aligned} {\left\{ \begin{array}{ll} \dot{\mathbf {x}}^+_j(t)=H_p(\mathbf {x}_j(t),p_j(t)) \\ H(\mathbf {x}_j(t),p_j(t))=\min _{p\in D^+u(\mathbf {x}_j(t))} H(\mathbf {x}_j(t),p) \end{array}\right. } \quad \forall t\in [0,\tau _j]\, (j=1,2) \end{aligned}$$

Therefore,

$$\begin{aligned} H_p(\mathbf {x}_1(t),p_1(t))=\phi '(t)H_p(\mathbf {x}_2(\phi (t)),p_2(\phi (t)))\quad ( t\in [0,\tau _1]) \end{aligned}$$

where, in addition to (3.24), we have that

$$\begin{aligned} p_2(\phi (t)))=\text{ arg }\min _{p\in D^+u(\mathbf {x}_2(\phi (t))} H(\mathbf {x}_2(\phi (t)),p) =\text{ arg }\min _{p\in D^+u(\mathbf {x}_1(t)} H(\mathbf {x}_1(t),p)=p_1(t). \end{aligned}$$

So, $H_p(\mathbf {x}_1(t),p_1(t))=\phi '(t)H_p(\mathbf {x}_1(t),p_1(t))$ for all $t\in [0,\tau _1]$. Since $0\not \in H_p(x_0,D^+u(x_0))$, we conclude that $\phi '(t)=1$, or $\phi (t)=t$, on some interval $0\leqslant t\leqslant \tau \leqslant \tau$.

Theorems 3.6 and 3.9 establish a connection between the absence of critical points and uniqueness of strict singular characteristics. In this direction, we also have the following global result.

### Corollary 3.10

Let *u* be a semiconcave solution of ($\hbox {HJ}_{\mathrm{loc}}$) and let $x_0\in \text{ Sing }\,(u)$. Let $\mathbf {x}_j:[0,T]\rightarrow \Omega$ ($j=1,2$) be strict singular characteristics with initial point $x_0$ such that $0\not \in H_p(\mathbf {x}_j(t),D^+u(\mathbf {x}_j(t)))$ for all $t\in [0,T]$. Then $\mathbf {x}_1(t)=\mathbf {x}_2(t)$ for all $t\in [0,T]$.

### Proof

On account of Theorem 3.9 we have that

$$\begin{aligned} \mathcal T:=\big \{\tau \in (0,T]~|~\mathbf {x}_1(t)=\mathbf {x}_2(t)\,,\;\forall t\in [0,\tau ]\big \} \end{aligned}$$

is a nonempty set. Let $\tau _0=\sup \mathcal T=\max \mathcal T$. We claim that $\tau _0=T$. For if $\tau _0<T$, applying Theorem 3.9 with initial point $\mathbf {x}_1(\tau _0)$ we conclude that $\mathbf {x}_1(t)=\mathbf {x}_2(t)$ on some intarval $\tau _0\leqslant t<\tau _0+\delta$, contradicting the definition of $\tau _0$.

Another well-known example where we have uniqueness of the generalized characteristic is the mechanical Hamiltonian

3.25 $$\begin{aligned} H(x,p)=\frac{1}{2}\langle A(x)p,p\rangle +V(x),\quad (x,p)\in \Omega \times \mathbb {R}^n, \end{aligned}$$

with *A*(*x*) is positive definite symmetric $n\times n$-matrix $C^2$-smooth in *x* and *V* a smooth function on $\Omega$. More precisely, if $x\in \text{ Sing }\,(u)$, then there exists a unique Lipschitz arc $\mathbf {y}$ determined by $\dot{\mathbf {y}}^+(t)=A(\mathbf {y}(t))p(t)$, where $\mathbf {y}(0)=x$ and $p(t)=\arg \min _{p\in D^+u(\mathbf {y}(t))}\langle A(\mathbf {y}(t))p,p\rangle$. In this case, uniqueness follows from semiconcavity by an application of Gronwall’s lemma (see, e.g., [2, 10]) ensuring that, in addition, any generalized characteristic is strict. We now give another justification of such a property from the point of view of this section.

### Corollary 3.11

If *H* is a mechanical Hamiltonian as in (3.25), then the reparameterization $\phi$ in Theorem 3.6 is the identity.

### Proof

We observe that, for almost all $t\geqslant 0$,

$$\begin{aligned} \dot{\mathbf {y}}(t)=A(\mathbf {y}(t))\{\lambda (t)p_0(t)+(1-\lambda (t))p_1(t)\} \end{aligned}$$

where $\lambda (t)\in [0,1]$ and we can assume $D^+u(\mathbf {y}(t))$ is a segment, say $[p_1(t),p_0(t)]$, or $\{p_0(t),p_1(t)\}\in D^*u(\mathbf {y}(t))$. Notice that $\{p_0(t),p_1(t)\}$ is also the set of extremal points of the convex set $D^+u(\mathbf {y}(t))$.

Since $\mathbf {x}(t)=\mathbf {y}(\phi (t))$, differentiating we obtain that

$$\begin{aligned} \dot{\mathbf {x}}(t)=p(t)=&\,\phi '(t)\dot{\mathbf {y}}(\phi (t))\\ =&\,\phi '(t)A(\mathbf {y}(\phi (t)))\{\lambda (\phi (t))p_0(\phi (t))+(1-\lambda (\phi (t)))p_1(\phi (t))\} \end{aligned}$$

with $$D^+u(\mathbf {y}(\phi (t)))=[p_0(\phi (t)),p_1(\phi (t))]$$, or $$\{p_0(\phi (t)),p_1(\phi (t))\}\in D^*u(\mathbf {y}(\phi (t)))$$.

Therefore, there exists a unique $\lambda _t\in [0,t]$ such that

$$\begin{aligned} p(t)=A(\mathbf {y}(\phi (t)))\{\lambda _tp_0(\phi (t))+(1-\lambda _t)p_1(\phi (t))\}. \end{aligned}$$

It follows that

$$\begin{aligned} \phi '(t)=\phi '(t)\{\lambda (\phi (t))+(1-\lambda (\phi (t))))=\lambda _t+(1-\lambda _t)=1. \end{aligned}$$

Thus, $\phi (t)\equiv t$ and this completes the proof.

### Remark 3.12

Observe that our results apply in particular to solutions of ($\hbox {HJ}_s$).

## Appendix A: Existence of strict singular characteristics

In this appendix, we prove the following result which ensures the existence of strict singular characteristics mentioned in the Introduction.

We recall that

$$\begin{aligned} \dot{\mathbf {x}}^+(t):=\lim _{h\downarrow 0}\frac{\mathbf {x}(t+h)-\mathbf {x}(t)}{h}\quad (t\in [0,T)) \end{aligned}$$

denotes the right derivative of $\mathbf {x}:[0,T]\rightarrow \Omega$, whenever such a derivative exists.

### Theorem A.1

Let *u* be a semiconcave solution of ($\hbox {HJ}_{\mathrm{loc}}$). If $x_0\in \text{ Sing }\,(u)$ satisfies

A.1 $$\begin{aligned} 0\not \in \text{ co } H_p(x_0,D^+u(x_0)), \end{aligned}$$

then there exists a Lipschitz singular arc $\mathbf {x}:[0,T]\rightarrow \Omega$ and a right-continuous selection $p(t)\in D^+u(\mathbf {x}(t))$ such that

A.2 $$\begin{aligned} \begin{aligned} {\left\{ \begin{array}{ll} \dot{\mathbf {x}}^+(t)=H_p(\mathbf {x}(t),p(t))&{} \forall t\in [0,T),\\ \mathbf {x}(0)=x_0.&{} \end{array}\right. } \end{aligned} \end{aligned}$$

and

A.3 $$\begin{aligned} H(\mathbf {x}(t),p(t))=\min _{p\in D^+u(\mathbf {x}(t))}H(\mathbf {x}(t),p)\quad \forall t\in [0,T). \end{aligned}$$

### Remark A.2

The existence of strict singular characteristics for time dependent Hamilton–Jacobi equations was proved by Khanin and Sobolevski under the additional assumption that the solution *u* can be locally represented as the minimum of a compact family of smooth functions. Theorem A.1 adapts [13, Theorem 2] to stationary equations removing such an extra assumption.

### Proof

The proof, which uses ideas from [13], requires several intermediate steps.

Let $R_0>0$ be such that the closed ball $B(x_0,2R_0)$ is contained in $\Omega$. Take any sequence of smooth functions $u_m:B(x_0,2R_0)\rightarrow \mathbb {R}$ such that

$$\begin{aligned} {\left\{ \begin{array}{ll} (a) &{} u_m{\mathop {\longrightarrow }\limits ^{m\rightarrow \infty }} u \;\; \text{ uniformly } \text{ on } B(x_0,R_0) \vspace{.1cm} \\ (b) &{} \max \{\Vert Du\Vert _{\infty }, \Vert Du_m\Vert _{\infty }\}\leqslant C_1 \\ (c)&{} D^2u_m\leqslant C_2I \end{array}\right. } \end{aligned}$$

for some constants $C_1,C_2>0$. A sequence with the above properties can be constructed in several ways, for instance by using mollifiers like in [11, 18]. In view of the above uniform bounds, there exists $T_0>0$ such that for any $m\geqslant 1$ the Cauchy problem

A.4 $$\begin{aligned} {\left\{ \begin{array}{ll} \dot{\mathbf {x}}(t)=H_p(\mathbf {x}(t),Du_m(\mathbf {x}(t))),&{}t\in [0,T_0]\\ \mathbf {x}(0)=x_0 \end{array}\right. } \end{aligned}$$

has a unique solution $\mathbf {x}_m:[0,T_0]\rightarrow B(x_0,R_0)$. Moreover, by possibly taking a subsequence, we can assume that $\mathbf {x}_m$ converges uniformly on $[0,T_0]$ to some Lipschitz arc $\mathbf {x}:[0,T_0]\rightarrow B(x_0,R_0)$. We will show that, after possibly replacing $T_0$ by a smaller $T>0$, such a limiting curve $\mathbf {x}$ has the required properties.

### Lemma A.3

For every $\bar{t}\in [0,T_0)$ and $\varepsilon >0$ there exists and integer $m_\varepsilon \geqslant 1$ and a real number $\tau _\varepsilon \in (0,T_0-\bar{t})$ such that

A.5 $$\begin{aligned} \frac{\mathbf {x}_m(t)-\mathbf {x}_m(\bar{t})}{t-\bar{t}}\in \text{ co } H_p\big (\mathbf {x}(\bar{t}),Du^+(\mathbf {x}(\bar{t}))\big )+\varepsilon B\quad \forall m\geqslant m_\varepsilon \,,\;\forall t\in [\bar{t},\bar{t}+\tau _\varepsilon ], \end{aligned}$$

where *B* denotes the closed unit ball of $\mathbb {R}^2$, centered at the origin.

### Proof

We begin by showing that for every $\bar{t}\in [0,T_0)$ and $\varepsilon >0$ there exist $m_\varepsilon \geqslant 1$ and $\tau _\varepsilon \in (0,T_0-\bar{t})$ satisfying

A.6 $$\begin{aligned} \dot{\mathbf {x}}_m(t)\in H_p\big (\mathbf {x}(\bar{t}),Du^+(\mathbf {x}(\bar{t}))\big )+\varepsilon B,\quad t\in [\bar{t},\bar{t}+\tau _\varepsilon ]\; \text{ a.e. } \end{aligned}$$

for all $m\geqslant m_\varepsilon$. We argue by contradiction: set $\Phi (\bar{t})= H_p\big (\mathbf {x}(\bar{t}),Du^+(\mathbf {x}(\bar{t}))\big )$ and suppose there exist $\bar{t}\in [0,T_0)$, $\varepsilon >0$, and sequences $m_k\rightarrow \infty$ and $t_k\downarrow \bar{t}$ such that

$$\begin{aligned} {\left\{ \begin{array}{ll} (i) &{} \dot{\mathbf {x}}_{m_k}(t_k)\notin \Phi (\bar{t})+\varepsilon B,\quad \forall k\geqslant 1 \\ (ii)&{}Du_{m_k}\big ( \mathbf {x}_{m_k}(t_k)\big )\rightarrow \bar{p} \quad (k\rightarrow \infty ) \end{array}\right. } \end{aligned}$$

where we have used bound (*b*) above to justify (*ii*). We claim that $\bar{p}\in D^+u\big (\mathbf {x}(\bar{t})\big )$. Indeed, in view of (*c*) above we have that, for all $k\geqslant 1$,

$$\begin{aligned} u_{m_k}\big ( \mathbf {x}_{m_k}(t_k)+y\big )-u_{m_k}\big ( \mathbf {x}_{m_k}(t_k)\big )-\big \langle Du_{m_k}\big ( \mathbf {x}_{m_k}(t_k)\big ),y\big \rangle \leqslant C_2|y|^2,\quad \forall |y|\leqslant R_0. \end{aligned}$$

Hence, in the limit as $k\rightarrow \infty$, we get

$$\begin{aligned} u( \mathbf {x}(\bar{t})+y)-u( \mathbf {x}(\bar{t}))-\langle \bar{p},y\rangle \leqslant C_2|y|^2,\quad \forall |y|\leqslant R_0, \end{aligned}$$

which in turn proves our claim. Thus, we conclude that

$$\begin{aligned} \dot{\mathbf {x}}_{m_k}(t_k)=H_p\big ( \mathbf {x}_{m_k}(t_k),Du_{m_k}( \mathbf {x}_{m_k}(t_k))\big ) {\mathop {\longrightarrow }\limits ^{k\rightarrow \infty }} H_p(\mathbf {x}(\bar{t}),\bar{p})\in \Phi (\bar{t}) \end{aligned}$$

in contrast with (*i*). So, (A.6) is proved.

Finally, (A.5) can be derived from (A.6) by integration.

By appealing to the upper semi-continuity of $D^+u$ and assumption (A.1) we conclude that there exists $T\in (0,T_0]$ such that

A.7 $$\begin{aligned} 0\notin \text{ co } H_p\big (\mathbf {x}( t),Du^+(\mathbf {x}( t))\big )\quad \forall t\in [0,T]. \end{aligned}$$

Now, fix any $\bar{t}\in [0,T)$ and let $\bar{v}\in \mathbb {R}^2$ be any vector such that

A.8 $$\begin{aligned} \lim _{j\rightarrow \infty }\frac{\mathbf {x}(\bar{t}+\tau _j)-\mathbf {x}(\bar{t})}{\tau _j}=\bar{v} \end{aligned}$$

for some sequence $\tau _j\searrow 0$ ($j\rightarrow \infty$). Observe that $\bar{v}\in \text{ co } H_p\big (\mathbf {x}(\bar{t}),Du^+(\mathbf {x}(\bar{t}))\big )$ in view of Lemma A.3. So, $\bar{v}\ne 0$ owing to (A.7). Set $\bar{x}=\mathbf {x}(\bar{t})$ and define

$$\begin{aligned}&\bar{p}\in \mathbb {R}^2 \quad \text{ by } \bar{v}=H_p(\bar{x},\bar{p}) \quad (\text{ or }\quad \bar{p}=L_v(\bar{x},\bar{v})) \\&F_{\bar{v}}(\bar{x})=\big \{p^*\in D^+u(\bar{x}):\langle p^*,\bar{v}\rangle =\min _{p\in D^+u(\bar{x})}\langle p,\bar{v}\rangle \big \}. \end{aligned}$$

Notice that $F_{\bar{v}}(\bar{x})$ is the exposed face of the convex set $D^+u(\bar{x})$ in the direction $\bar{v}$ (see, for instance, [10]). The following lemma identifies $\bar{p}$ (hence $\bar{v}$) uniquely.

### Lemma A.4

Suppose $\bar{p}\in F_{\bar{v}}(\bar{x})$. Then $\bar{p}$ is the unique element in $D^+u(\bar{x})$ such that

A.9 $$\begin{aligned} H(\bar{x},\bar{p})=\min _{p\in D^+u(\bar{x})}H(\bar{x},p). \end{aligned}$$

### Proof

Since $\bar{p}\in F_{\bar{v}}(\bar{x})$, we have that

$$\begin{aligned} \langle \bar{p},\bar{v}\rangle =\langle \bar{p},H_p(\bar{x},\bar{p})\rangle =\min _{p\in D^+u(\bar{x})}\langle p, H_p(\bar{x},\bar{p})\rangle . \end{aligned}$$

Therefore, by convexity we conclude that

$$\begin{aligned} 0\leqslant \langle H_p(\bar{x},\bar{p}),p-\bar{p}\rangle \leqslant H(\bar{x},p)-H(\bar{x},\bar{p}),\quad \forall p\in D^+u(\bar{x}). \end{aligned}$$

Since *H* is strictly convex in *p*, $\bar{p}$ is the unique element in $D^+u(\bar{x})$ satisfying (A.9).

Notice that the above lemma yields the existence of the right-derivative $\dot{\mathbf {x}}^+(\bar{t})$ as soon as one shows that $\bar{p}\in F_{\bar{v}}(\bar{x})$ for any $\bar{v}$ satisfying (A.8).

Next, to show that $\bar{p}\in F_{\bar{v}}(\bar{x})$, we proceed by contradiction assuming that

A.10 $$\begin{aligned} \bar{p}\not \in F_{\bar{v}}(\bar{x}). \end{aligned}$$

Let us define functions $\alpha ,\beta :D^+u(\bar{x})\rightarrow \mathbb {R}$ by

$$\begin{aligned} \alpha (p)=\langle p,\bar{v}\rangle -\frac{\partial u}{\partial \bar{v}}(\bar{x}),\quad \beta (x,p)= \langle p-\bar{p},H_p(x,p)-H_p(x,\bar{p})\rangle \quad \forall p\in D^+u(\bar{x}) \end{aligned}$$

where we have set $\frac{\partial u}{\partial \bar{v}}(\bar{x})=\lim _{\lambda \rightarrow 0^+}\frac{u(\bar{x}+\lambda \bar{v})-u(\bar{x})}{\lambda }$. Recall that, since *u* is semiconcave,

A.11 $$\begin{aligned} \frac{\partial u}{\partial \bar{v}}(\bar{x})=\min _{p\in D^+u(\bar{x})}\langle p,\bar{v}\rangle \end{aligned}$$

(see, for instance, [10]). The following simple lemma is crucial for the proof.

### Lemma A.5

If $$\bar{p}\not \in F_{\bar{v}}(\bar{x})$$, then

$$\begin{aligned} \mu :=\min _{p\in D^+u(\bar{x})}\{\alpha (p)+\beta (\bar{x},p)\}>0. \end{aligned}$$

### Proof

Observe first that $\beta (x,p)\geqslant 0$ by convexity and $\alpha (p)\geqslant 0$ for all $p\in D^+u(\bar{x})$ by (A.11). Since we suppose $\bar{p}\not \in F_{\bar{v}}(\bar{x})$, just two cases are possible.

1. If $\bar{p}\not \in D^+u(\bar{x})$, then $p\not =\bar{p}$ for all $p\in D^+u(\bar{x})$. So $\beta (\bar{x},p)>0$ by strict convexity.
2. If $$\bar{p}\in D^+u(\bar{x})\setminus F_{\bar{v}}(\bar{x})$$, then $$\alpha (p)>0$$.

In conclusion,

$$\begin{aligned} M(p):=\alpha (p)+\beta (\bar{x},p)>0,\quad \forall p\in D^+u(\bar{x}). \end{aligned}$$

Since *M* is continuous and $D^+u(\bar{x})$ is compact, the conclusion follows.

For any $\varepsilon >0$ set

$$\begin{aligned} F^{\varepsilon }_{\bar{v}}(\bar{x})=F_{\bar{v}}(\bar{x})+\varepsilon B\quad \text{ and }\quad V_{\varepsilon }=D^+u(\bar{x})+\varepsilon B. \end{aligned}$$

Now, let us fix $\varepsilon =\varepsilon (\bar{v},\mu )>0$ such that

A.12 $$\begin{aligned} \bar{p}\not \in F^{\varepsilon }_{\bar{v}}(\bar{x})\quad \text {and} \quad \min _{p\in V_{\varepsilon }}\{\alpha (p)+\beta (\bar{x},p)\}\geqslant \frac{2}{3}\mu . \end{aligned}$$

Let $0<R\leqslant R_0$ be such that

$$\begin{aligned} D^+u(x)\subset V_{\varepsilon /2}\quad \forall x\in B(\bar{x},R). \end{aligned}$$

Consider the line segment

$$\begin{aligned} \gamma (t):=\bar{x}+(t-\bar{t})\bar{v}\quad (t\in [\bar{t},T]) \end{aligned}$$

and fix $q\in (0,1)$. After possible reducing *T*, we can assume that

$$\begin{aligned} |\gamma (t)-\bar{x}|\leqslant qR\quad \text {and}\quad |\mathbf {x}(t)-\bar{x}|\leqslant qR\quad \forall t\in [\bar{t},T]. \end{aligned}$$

Consequently, there exists $\bar{m}\in \mathbb {N}$ such that for all $m\geqslant \bar{m}$ we have

(i) $$Du_m(x)\in V_{\varepsilon }$$ for all $$x\in B(\bar{x},R)$$;
(ii) $\mathbf {x}_m(t)\in B(\bar{x},R)$ for all $t\in [\bar{t},T]$.

Moreover, by cutting *T* down to size, we can have the following property satisfied:

(iii) for any $t\in [\bar{t},T]$ there exists $m(t)\geqslant \bar{m}$ such that
  A.13 $$\begin{aligned} d_{F_{\bar{v}}(\bar{x})}(Du_m(\gamma (t)))<\varepsilon ,\quad \forall m\geqslant m(t). \end{aligned}$$

We observe that (iii) is a consequence of Proposition 3.3.15 in [10] since $\bar{v}\not =0$.

For $0<\delta$ to be chosen later on, we define

$$\begin{aligned} K_{\delta }=&\,\bigcup _{\bar{t}\leqslant t\leqslant T}B(\gamma (t),\delta (t-\bar{t}))\\ =&\,\{x\in \mathbb {R}^n: \text {there exists}\ t\in [\bar{t},T]\ \text {such that}\ |x-\gamma (t)|\leqslant \delta (t-\bar{t})\}. \end{aligned}$$

### Lemma A.6

Let $\varepsilon >0$ and $m(\cdot )$ be fixed so that (A.12) and (A.13) hold true. If $\bar{p}\not \in F_{\bar{v}}(\bar{x})$, then there exists $\delta >0$ such that for all *j* sufficiently large, $\mathbf {x}_m(t)\not \in K_{\delta }$ for all $t\in (\bar{t}+3\tau _j,T)$ and *m* sufficiently large.

### Proof

Throughout this proof $j\in \mathbb {N}$ is supposed to be so large that $\tau _j<(T-\bar{t})/3$. Moreover, in order to simplify the notation, abbreviate $\tau$ for $\tau _j$ and we assume $\bar{t}=0$.

For all $t\in (3\tau ,T)$ we have that

$$\begin{aligned}&\,\frac{d}{dt}\big (u_m(\mathbf {x}_m(t))-\langle \bar{p},\mathbf {x}_m(t) \rangle \big )\\&\quad =\,\big \langle Du_m(\mathbf {x}_m(t))-\bar{p},\dot{\mathbf {x}}_m(t) \big \rangle =\big \langle H_p(\mathbf {x}_m(t),Du_m(\mathbf {x}_m(t))),Du_m(\mathbf {x}_m(t))-\bar{p}\big \rangle . \end{aligned}$$

Therefore, by integrating on $(\tau ,t)$,

A.14 $$\begin{aligned} \begin{aligned}&\,u_m(\mathbf {x}_m(t))-\langle \bar{p},\mathbf {x}_m(t)\rangle -u_m(\mathbf {x}_m(\tau ))+\langle \bar{p},\mathbf {x}_m(\tau )\rangle \\&\quad =\,\int ^t_{\tau } \big \langle H_p(\mathbf {x}_m(s),Du_m(\mathbf {x}_m(s))), Du_m(\mathbf {x}_m(s))-\bar{p}\big \rangle ds. \end{aligned} \end{aligned}$$

Similarly,

$$\begin{aligned} \frac{d}{dt}\big (u_m(\gamma (t)))- \langle \bar{p},\gamma (t)\rangle \big ) = \langle Du_m(\gamma (t))-\bar{p},\bar{v}\rangle . \end{aligned}$$

So, (iii) and Lebesgue’s theorem ensure that

A.15 $$\begin{aligned} \begin{aligned}&\,u_m(\gamma (t))- \langle \bar{p},\gamma (t) \rangle -u_m(\gamma (\tau ))+ \langle \bar{p},\gamma (\tau ) \rangle \\&\quad =\, \int ^t_{\tau } \langle Du_m(\gamma (s))-\bar{p},\bar{v}\rangle \ ds \leqslant \bigg (\frac{\partial u}{\partial \bar{v}}(\bar{x})- \langle \bar{p},\bar{v}\rangle +\varepsilon |\bar{v}|\bigg )(t-\tau ). \end{aligned} \end{aligned}$$

Therefore, by (A.14) and (A.15) we obtain

$$\begin{aligned}&\,u_m(\mathbf {x}_m(t))-\langle \bar{p},\mathbf {x}_m(t)\rangle -u_m(\mathbf {x}_m(\tau ))+\langle \bar{p},\mathbf {x}_m(\tau )\rangle \\&\qquad \,-\big (\,u_m(\gamma (t))- \langle \bar{p},\gamma (t) \rangle -u_m(\gamma (\tau ))+ \langle \bar{p},\gamma (\tau ) \rangle \big )\\&\quad \geqslant \,\int ^t_{\tau }\bigg \{\big \langle H_p(\mathbf {x}_m(s),Du_m(\mathbf {x}_m(s))), Du_m(\mathbf {x}_m(s))-\bar{p}\big \rangle -\bigg (\frac{\partial u}{\partial \bar{v}}(\bar{x})- \langle \bar{p},\bar{v}\rangle +\varepsilon |\bar{v}|\bigg )\bigg \}ds \end{aligned}$$

which can be rewritten as

A.16 $$\begin{aligned}&\,\,u_m(\mathbf {x}_m(t))-\langle \bar{p},\mathbf {x}_m(t)\rangle -u_m(\mathbf {x}_m(\tau ))+\langle \bar{p},\mathbf {x}_m(\tau )\rangle \nonumber \\&\qquad \,-\big (\,u_m(\gamma (t))- \langle \bar{p},\gamma (t) \rangle -u_m(\gamma (\tau ))+ \langle \bar{p},\gamma (\tau ) \rangle \big )\nonumber \\&\quad \geqslant \, \int ^t_{\tau } \big \langle H_p(\mathbf {x}_m(s),Du_m(\mathbf {x}_m(s)))-\bar{v}, Du_m(\mathbf {x}_m(s))-\bar{p}\big \rangle ds\nonumber \\&\qquad \,+\int ^t_{\tau }\bigg (\langle Du_m(\mathbf {x}_m(s)),\bar{v}\rangle -\frac{\partial u}{\partial \bar{v}}(\bar{x})-\varepsilon |\bar{v}|\bigg )\ ds\nonumber \\&\quad =\, \int ^t_{\tau } \big \langle H_p(\mathbf {x}_m(s),Du_m(\mathbf {x}_m(s)))-H_p(\mathbf {x}_m(s), \bar{p}),Du_m(\mathbf {x}_m(s))-\bar{p}\big \rangle ds\nonumber \\&\qquad \,+\int ^t_{\tau }\big \langle H_p(\mathbf {x}_m(s),\bar{p})-H_p(\bar{x},\bar{p}),Du_m(\mathbf {x}_m(s))-\bar{p}\big \rangle \ ds\nonumber \\&\qquad \,+\int ^t_{\tau }\bigg (\langle Du_m(\mathbf {x}_m(s)),\bar{v}\rangle -\frac{\partial u}{\partial \bar{v}}(\bar{x})-\varepsilon |\bar{v}|\bigg )\ ds\nonumber \\&\quad \geqslant \,\int ^t_{\tau }\big \{\alpha (Du_m(\mathbf {x}_m(s)))+\beta (\mathbf {x}_m(s),Du_m(\mathbf {x}_m(s)))-\varepsilon |\bar{v}|\big \} ds\nonumber \\&\qquad \,+\int ^t_{\tau }\big \langle H_p(\mathbf {x}_m(s),\bar{p})-H_p(\bar{x},\bar{p}),Du_m(\mathbf {x}_m(s))-\bar{p}\big \rangle \ ds . \end{aligned}$$

Now, observe the following:

$$\begin{aligned}&|u_m(\gamma (t))- \langle \bar{p},\gamma (t) \rangle -u_m(\gamma (\tau ))+ \langle \bar{p},\gamma (\tau ) \rangle | \leqslant \,(C_1+|\bar{p}|)|\gamma (\tau )-\mathbf {x}_m(\tau )|\\&\quad \leqslant \,(C_1+|\bar{p}|)(|\gamma (\tau )-\mathbf {x}(\tau )|+|\mathbf {x}(\tau )-\mathbf {x}_m(\tau )|) \end{aligned}$$

where we recall that $C_1\geqslant \Vert Du_m\Vert _{\infty }$.

Next, we fix $\tau =\tau _j$ with *j* large enough so that

$$\begin{aligned} |\gamma (\tau )-\mathbf {x}(\tau )|\leqslant \frac{\delta \tau }{2}\quad \text {and}\quad m\gg 1\ \text {so that}\ |\mathbf {x}(\tau )-\mathbf {x}_m(\tau )|\leqslant \frac{\delta \tau }{2}. \end{aligned}$$

Then

A.17 $$\begin{aligned} |u_m(\gamma (\tau ))- \langle \bar{p},\gamma (\tau ) \rangle -u_m(\mathbf {x}_m(\tau ))+ \langle \bar{p},\mathbf {x}_m(\tau ) \rangle | \leqslant \,(C_1+|\bar{p}|)\delta \tau . \end{aligned}$$

Since $Du_m(\mathbf {x}_m(s))\in V_{\varepsilon }$ for all $s\in [0,\tau ]$, by (A.12) we have that

A.18 $$\begin{aligned} \int ^t_{\tau }\bigg \{\alpha (Du_m(\mathbf {x}_m(s)))+\beta (\mathbf {x}_m(s),Du_m(\mathbf {x}_m(s)))\bigg \} ds\geqslant \frac{2}{3}\mu (t-\tau ). \end{aligned}$$

We also have that, after cutting down on $T>0$,

A.19 $$\begin{aligned} \begin{aligned}&\, \langle H_p(\mathbf {x}_m(s),\bar{p})-H_p(\bar{x},\bar{p}),Du_m(\mathbf {x}_m(s))-\bar{p} \rangle \\&\quad \geqslant \,-(C_1+|\bar{p}|)\cdot C'_2|\mathbf {x}_m(s)-\bar{x}|\\&\quad \geqslant \,-\varepsilon C'_2(C_1+|\bar{p}|) \end{aligned} \end{aligned}$$

So, by (A.16), (A.17), (A.18) and (A.19) we conclude that

$$\begin{aligned}&\,u_m(\mathbf {x}_m(t))- \langle \bar{p},\mathbf {x}_m(t)\rangle -u_m(\gamma (t))+ \langle \bar{p},\gamma (t)\rangle \\&\quad \geqslant \,\bigg (\frac{2}{3}\mu -\varepsilon (|\bar{v}|+C'_2(C_1+|\bar{p}|))\bigg )(t-\tau )-\delta \tau (C_1+|\bar{p}|). \end{aligned}$$

On the other hand,

$$\begin{aligned} |u_m(\mathbf {x}_m(t))- \langle \bar{p},\mathbf {x}_m(t)\rangle -u_m(\gamma (t))+ \langle \bar{p},\gamma (t)\rangle |\leqslant (C_1+|\bar{p}|)|\mathbf {x}_m(t)-\gamma (t)|. \end{aligned}$$

Therefore,

A.20 $$\begin{aligned} |\mathbf {x}_m(t)-\gamma (t)|\geqslant \frac{2\mu /3-\varepsilon (|\bar{v}|+C'_2(C_1+|\bar{p}|))}{C_1+|\bar{p}|}(t-\tau )-\delta \tau . \end{aligned}$$

We now take $0\leqslant \varepsilon (|\bar{v}|+C'_2(C_1+|\bar{p}|))|<\frac{\mu }{3}$ to obtain

$$\begin{aligned} |\mathbf {x}_m(t)-\gamma (t)|\geqslant \frac{\mu (t-\tau )}{3(C_1+|\bar{p}|)}-\delta \tau \end{aligned}$$

and look for $t<T$ such that

A.21 $$\begin{aligned} \frac{\mu (t-\tau )}{3(C_1+|\bar{p}|)}-\delta \tau \geqslant 2\delta t, \end{aligned}$$

or

$$\begin{aligned} t\geqslant \frac{3(C_1+|\bar{p}|)\delta +\mu }{\mu -6(C_1+|\bar{p}|)\delta }\cdot \tau . \end{aligned}$$

So, taking $0<\delta \leqslant \frac{\mu }{12(C_1+|\bar{p}|)}$, we have that

$$\begin{aligned} \frac{3(C_1+|\bar{p}|)\delta +\mu }{\mu -6\delta (C_1+|\bar{p}|)}\leqslant 3. \end{aligned}$$

Finally, $\frac{\delta }{\mu }\leqslant \frac{1}{12(C_1+|\bar{p}|)}$ gives that (A.21) holds for all $t\in [3\tau ,T]$. $\square$

To complete the proof it suffices to note that Lemmas A.5 and A.6 ensure that assuming (A.10) leads to a contradiction. Indeed,

$$\begin{aligned} |\mathbf {x}_m(t)-\gamma (t)|\geqslant 2\delta t,\quad \forall t\in [3\tau _j,T], \forall j\gg 1 \end{aligned}$$

implies that $\mathbf {x}(t)\not \in K_{\delta }$ for all $t\in [3\tau _j,T]$. On the other hand, $\mathbf {x}(\tau _i)\in K_{\delta }$ for $i\gg 1$ and, for any fixed *i*, $\tau _i\in [3\tau _j,T]$ for *j* sufficiently large.

## Footnotes

1 This is the point where our reasoning requires to be in dimension 2.
